# Pediatric Hydatid Cyst of the Neck Mimicking a Cystic Tumor: A Report of a Rare Case

**DOI:** 10.7759/cureus.37328

**Published:** 2023-04-09

**Authors:** Yassine Ait M'barek, Hajar Hamadi, Lamia Benantar, Tariq Belokda, Elmehdi Hamidi, Khalid Aniba

**Affiliations:** 1 Neurological Surgery, Ibn Tofail Hospital, Mohammed VI University Hospital, Marrakech, MAR

**Keywords:** echinococcus granulosis, hydatidosis, neck mass, surgery, neck, hydatid cyst

## Abstract

Hydatidosis is a parasitic infection caused by the cestode *Echinococcus granulosus *usually occurring in the liver and lungs. Hydatid cyst of the neck is a rarely described location and more so on the back of the neck. We present a case of a six-year-old girl with a slowly evolving mass on the back of her neck. Medical investigations revealed a secondary asymptomatic liver cyst. MRI of the neck mass was consistent with a cystic lesion. Surgical removal of the neck cyst was performed. Pathological examination results confirmed the diagnosis of hydatid cyst. The patient was put on medical treatment with a complete recovery and uneventful follow-up.

## Introduction

Hydatidosis or echinococcosis is a zoonotic infection caused by the cestode *Echinococcus granulosus*. Dog is the intended host, but the infection can occur in intermediate hosts such as sheep, cattle, and horses [1-3]. As a consequence, hydatid disease is endemic in sheep and cattle-rearing regions, like Australia, New Zealand, South America, East Africa, Central Europe, the Middle East, and the Mediterranean countries [3-5]. Humans are accidental intermediate hosts and contract the infection through the ingestion of the parasite’s eggs [1,3]. The occurrence of hydatid cysts in the head and neck is extremely rare even in countries where *Echinococcus* infestation is endemic [2,4].

With only seven cases of hydatid cysts of the back of the neck reported in the current literature, our case would help highlight the importance of recalling hydatid cysts among plausible differential diagnoses of cervical masses.

## Case presentation

A six-year-old girl living in the countryside was admitted to the Department of Neurosurgery with a painless, slowly growing mass located in the neck, evolving for almost two years. The patient had no significant personal or family history and no contact history with dogs, but she was living in a rural area. Physical examination revealed a 4x5cm painless non-tender mass located on the midline of the suboccipital area. The mass was covered with healthy skin, non-reductible with a soft consistency, and non-adherent to the adjacent neck muscles. There were no signs of local inflammation. Abdominal and pulmonary examinations were normal and biological tests were within normal limits.

**Figure 1:**
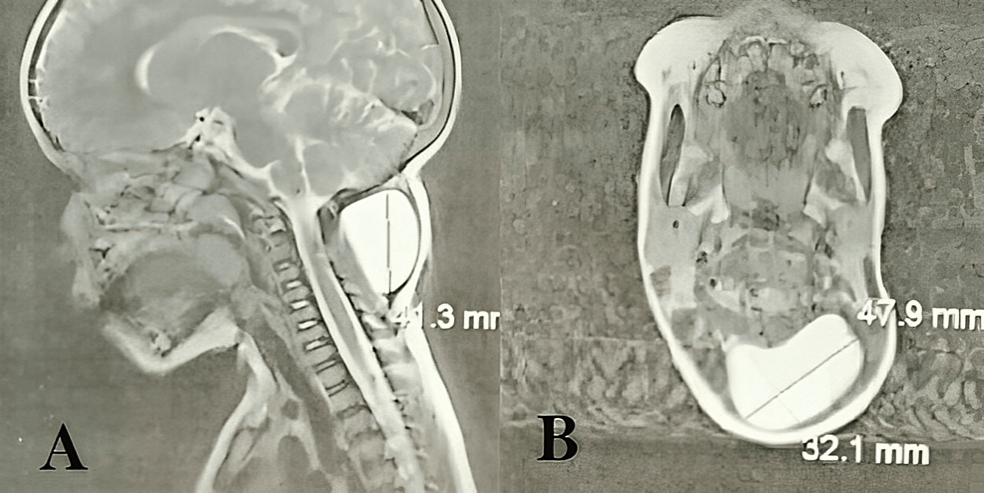
Cervical MRI of the neck cyst A: Cervical MRI in T2-weighted sequence sagittal plane showing a well-demarcated cystic lesion posterior to the vertebrae, extending from C1 to C5 with no apparent invasion of the adjacent tissue. B: Cervical MRI in T2-weighted sequence axial plane showing the same well-demarcated cystic lesion.

Cervical magnetic resonance imaging (MRI) showed an encapsulated cystic mass with no septations, and no invasion of adjacent tissues and organs (Figure 1). At this point, the differential diagnosis included cystic lymphangioma, cold abscess, and epidermal cyst.

Further investigations were performed to search for other locations. The abdominal ultrasonogram found a solitary asymptomatic hepatic cyst type 1 (according to Gharbi's classification) of the right posterior segment measuring 19x17.5x21.2 mm. Chest X-ray and CT scan of the brain were normal, and indirect hemagglutination for hydatid disease was negative. A stool examination was not performed.

**Figure 2:**
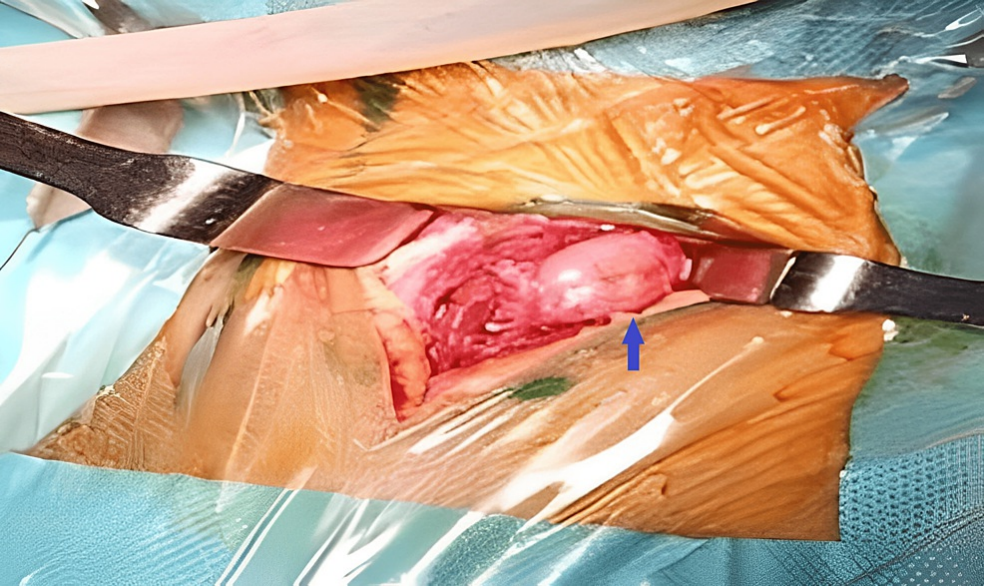
Peri-operative image of the surgical site showing the ruptured hydatid cyst wall (blue arrow).

The patient underwent total removal of the cyst under general anesthesia. Incision of the skin was followed by sharp careful dissection of soft tissues (Figure 2). During the procedure, the characteristic capsule of the cyst was accidentally ruptured releasing a clear water-like fluid. Once the cyst wall was fully removed, the surgical site was filled with a hypertonic serum to avoid anaphylactic shock following the total removal of the cyst. Post-operative follow-up was uneventful and the patient was immediately started on albendazole.

**Figure 3:**
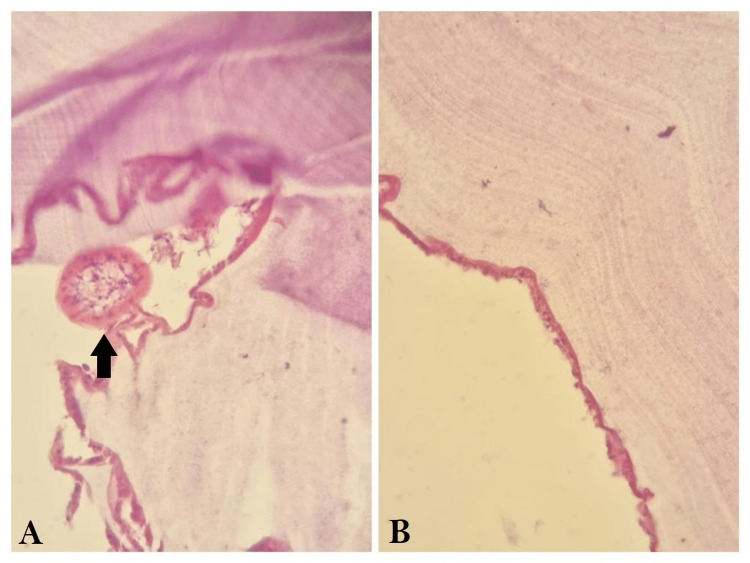
Histopathological examination of the cyst after resection. A: Image showing a cross-section of a scolex (black arrow). B: Image showing the cyst wall and its characteristic laminated membrane which is lined by a germinal epithelium.

Pathological examination confirmed the diagnosis of a hydatid cyst (Figure 3). The patient underwent a four-month course of albendazole at the recommended dose of 10mg/kg twice a day and was referred to a pediatric surgeon for the management of the hepatic cyst.

The patient was seen on the six-months follow-up and showed no recurrence or secondary locations. The hepatic cyst was surgically removed and the post-operative follow-up was uneventful.

## Discussion

Hydatid cyst is a zoonosis due to the development of *Echinococcus granulosus* tapeworm larvae in human tissue [2,3,6,7]. It is a parasitic infection endemic in most developing countries [2,8]. Hydatid cyst is endemic in Morocco representing a serious public health problem despite active policies to screen, diagnose, and treat the disease. Definitive hosts are dogs, foxes, and jackals, which explains the high incidence of the disease in traditional farming areas [2,3]. Humans accidentally host the infection after ingesting contaminated food or water or after direct contact with the definitive hosts [1,3,6,9].

The most common sites of occurrence are the liver (65-75%) and lungs (15-25%) in humans but it can occur in any location [2,3,10]. According to the literature, head and neck hydatidosis is extremely rare even in endemic areas, accounting for about 1% of all cases [3,10-12]. Furthermore, only a few cases of isolated hydatid cyst of the neck have been reported [2,6,11].

The diagnosis is rarely established in the presence of an isolated neck mass [2,9]. In our case, there was a simultaneous asymptomatic liver cyst revealed only after systematic biological and radiological investigations were performed screening for other potential locations.

Hydatidosis of the neck affects all ages with a sex ratio of 1, but studies show a clear predominance amongst the pediatric population [1]. It is usually asymptomatic with clinical features limited to the incidental discovery of a slowly growing, painless, non-inflammatory mass of the neck [2,3,7]. In some cases, the swelling can be responsible for the compression of adjacent organs, resulting in symptoms of dyspnea or dysphagia [2,3,10]. Due to the lack of characteristic clinical findings, other differential diagnoses can still be suspected including cystic lymphangioma, cold abscess, chronic hematoma, bronchial cleft cyst, thyroglossal duct cyst, and epidermal cyst [13]. 

Ultrasound of the mass remains the exam of choice, showing hydatid sands in a purely cystic lesion as well as floating membranes, daughter cysts, and vesicles [10,11,14]. MRI on the other hand remains more specific, allowing for a better anatomical and structural study of the lesion and its surroundings depicting signs of compression [14,15]. In our case, cervical MRI was inconclusive, showing an isolated cervical cyst in hyperintense signal on T1 sequence and an enhancement of the capsule after gadolinium injection. Aspiration cytology use is controversial because it can lead to the dissemination of daughter cysts or precipitate anaphylactic reactions [7]. Relevant studies have proved that aspiration cytology is responsible for a 1% risk of dissemination and anaphylactic shock [2]. Therefore, we advise that when confronted with a cystic lesion of the cervical region, the diagnosis of a hydatid cyst should be raised and biopsy should preferably be avoided before surgery to reduce this risk [14]. If the diagnosis remains doubtful, pathology confirms the diagnosis through the identification of a cyst wall composed of an outer acellular membrane and an inner nucleated germinal membrane, along with scolexes in some cases [2,6,14]. Serology can confirm the diagnosis in 80-90% of cases; nevertheless, negative results do not exclude hydatid disease as a potential diagnosis [2,6].

Definitive treatment of hydatid cysts is surgery based on a complete en-bloc surgical resection of the cyst [2,10,14]. Subtotal pericystectomy or resection of the prominent dome should only be performed if the cyst is adherent to the surrounding structures [2]. In case of rupture, the surgical site must be thoroughly sterilized using hypertonic saline solution or hydrogen peroxide to reduce the risk of dissemination [1,2,10].

Further explorations are systematic in the presence of a hydatid cyst of the neck. Abdominopelvic ultrasound and chest x-ray may confirm the presence of other locations [16]. Medical treatment with albendazole or mebendazole is indicated as an adjuvant therapy in case of peroperative rupture of the cyst or as a treatment in case of hydatidosis in inoperable and multiple locations [2,10,14]. Therapy with imidazole derivatives can also be useful to reduce the risk of recurrence [2,10,14].

Postoperative follow-up is usually uneventful if the surgical removal of the cyst is done properly; nevertheless, postoperative immediate and long-term follow-up is advised consisting of neck ultrasounds and hydatid serology [2,6].

## Conclusions

Hydatid cyst of the neck is a rare condition caused by an infestation of the larvae of the *Echinococcus granulosus*. The cyst grows slowly and can compress blood vessels and nerves causing pain, difficulty swallowing or breathing, and other symptoms. The positive diagnosis is not always straightforward, especially in slow-growing neck masses in the pediatric population. Treatment involves surgical removal of the cyst followed by medication to prevent recurrence. Monitoring for potential complications during surgery, such as rupture or dissemination is a crucial step. Early detection and prompt treatment are essential to prevent further damage to surrounding tissues and organs as well as reduce the incidence of complications.

## References

[1] Assamadi M, Benantar L, Hamadi H, Ksiks O, El Hadwe S, Aniba K (2022). Cerebral hydatid cyst in children: a case series of 21 patients and review of literature. Neurochirurgie.

[2] Oqbani K, Chraïbi M, Harchichi N, Abbaoui S (2017). Primary hydatid cyst of the neck: a rare and unusual site. Eur Ann Otorhinolaryngol Head Neck Dis.

[3] Goyal P, Ghosh S, Sehgal S, Panda I, Kumar A, Singh S, Tangri NK (2014). Primary multilocular hydatid cyst of neck with unique presentation: a rare case report and literature review. Head Neck Pathol.

[4] Eroğlu A, Atabekoğlu S, Kocaoğlu H (1999). Primary hydatid cyst of the neck. Eur Arch Otorhinolaryngol.

[5] Unal AE, Ulukent SC, Bayar S, Demirkan A, Akgül H (2001). Primary hydatid cyst of the axillary region: report of a case. Surg Today.

[6] Vecchio R, Marchese S, Ferla F, Spataro L, Intagliata E (2013). Solitary subcutaneous hydatid cyst: review of the literature and report of a new case in the deltoid region. Parasitol Int.

[7] Katilmiş H, Oztürkcan S, Ozdemir I, Adadan Güvenç I, Ozturan S (2007). Primary hydatid cyst of the neck. Am J Otolaryngol.

[8] Chakrabarti I, Goswami BK (2012). Primary hydatid cyst of the neck diagnosed by aspiration cytology. Trop Parasitol.

[9] Acharya S, Panda S, Biswal S (2022). Primary hydatid cyst of neck, a rare case report. Indian J Otolaryngol Head Neck Surg.

[10] El Bousaadani A, Abada R, Rouadi S, Roubal M, Mahtar M, Kadiri F (2016). Head and neck localizations of hydatid cyst: a series of 17 cases (Article in French). Rev Stomatol Chir Maxillofac Chir Orale.

[11] Khare P, Kala P, Gupta R, Chauhan N (2014). Isolated Echinococcosis of cervical region. J Cytol.

[12] Versaci A, Scuderi G, Rosato A (2005). Rare localizations of echinococcosis: personal experience. ANZ J Surg.

[13] Iranpour P, Masroori A (2018). Hydatid cyst of the neck mimicking a branchial cleft cyst. BMJ Case Rep.

[14] Karaman E, Yilmaz M, Ada M, Yilmaz RS, Isildak H (2011). Unusual location of primary hydatid cyst: soft tissue mass in the parapharyngeal region. Dysphagia.

[15] Alouini Mekki R, Mhiri Souei M, Allani M (2005). Hydatid cyst of soft tissues: contribution of MRI (about three cases) (Article in French). Journal de Radiologie.

[16] Hmidi M, Touiheme N, Rbai M, Messary A (2012). Isolated hydatid cyst of the neck: an unusual site. Eur Ann Otorhinolaryngol Head Neck Dis.

